# A critical analysis of test-retest reliability in instrument validation studies of cancer patients under palliative care: a systematic review

**DOI:** 10.1186/1471-2288-14-8

**Published:** 2014-01-21

**Authors:** Carlos Eduardo Paiva, Eliane Marçon Barroso, Estela Cristina Carneseca, Cristiano de Pádua Souza, Felipe Thomé dos Santos, Rossana Verónica Mendoza López, Sakamoto Bianca Ribeiro Paiva

**Affiliations:** 1 Department of Clinical Oncology, Barretos Cancer Hospital, Barretos, São Paulo CEP 14784-400, Brazil; 2 Palliative Care and Quality of Life Research Group, Post-Graduate Program, Barretos Cancer Hospital, Barretos, São Paulo, Brazil; 3 Researcher Support Center, Learning and Research Institute, Barretos Cancer Hospital, Barretos, São Paulo, Brazil

**Keywords:** Validation studies, Test-retest reliability, Systematic review, Cancer, Palliative care

## Abstract

**Background:**

Patient-reported outcome validation needs to achieve validity and reliability standards. Among reliability analysis parameters, test-retest reliability is an important psychometric property. Retested patients must be in a clinically stable condition. This is particularly problematic in palliative care (PC) settings because advanced cancer patients are prone to a faster rate of clinical deterioration. The aim of this study was to evaluate the methods by which multi-symptom and health-related qualities of life (HRQoL) based on patient-reported outcomes (PROs) have been validated in oncological PC settings with regards to test-retest reliability.

**Methods:**

A systematic search of PubMed (1966 to June 2013), EMBASE (1980 to June 2013), PsychInfo (1806 to June 2013), CINAHL (1980 to June 2013), and SCIELO (1998 to June 2013), and specific PRO databases was performed. Studies were included if they described a set of validation studies. Studies were included if they described a set of validation studies for an instrument developed to measure multi-symptom or multidimensional HRQoL in advanced cancer patients under PC. The COSMIN checklist was used to rate the methodological quality of the study designs.

**Results:**

We identified 89 validation studies from 746 potentially relevant articles. From those 89 articles, 31 measured test-retest reliability and were included in this review. Upon critical analysis of the overall quality of the criteria used to determine the test-retest reliability, 6 (19.4%), 17 (54.8%), and 8 (25.8%) of these articles were rated as good, fair, or poor, respectively, and no article was classified as excellent. Multi-symptom instruments were retested over a shortened interval when compared to the HRQoL instruments (median values 24 hours and 168 hours, respectively; p = 0.001). Validation studies that included objective confirmation of clinical stability in their design yielded better results for the test-retest analysis with regard to both pain and global HRQoL scores (p < 0.05). The quality of the statistical analysis and its description were of great concern.

**Conclusion:**

Test-retest reliability has been infrequently and poorly evaluated. The confirmation of clinical stability was an important factor in our analysis, and we suggest that special attention be focused on clinical stability when designing a PRO validation study that includes advanced cancer patients under PC.

## Background

Advanced cancer patients under palliative care (PC) experience many physical, psychosocial, and existential problems [1]. The PC team is essential for the screening, diagnosis, and treatment of cancer symptoms with the aim of improving the patients’ health-related quality of life (HRQoL). Therefore, an ideal assessment of symptoms and HRQoL should be performed using validated patient-reported outcome (PRO) instruments [2].

The process of PRO validation requires time and includes rigorous methods of data analysis. It encompasses the translation of foreign languages, cultural adaptation, and the evaluation of psychometric properties. Overall, a PRO validation needs to achieve the standards of validity and reliability [2]. Among reliability analyses, the most important are internal consistency, inter-rater reliability, and test-retest reliability. Test-retest reliability can be defined as “a measure of the reproducibility of the scale, that is, the ability to provide consistent scores over time in a stable population” [3].

Retested patients must be in a stable condition with respect to the construct to be measured by the PRO. This situation is particularly problematic in PC settings because advanced cancer patients are prone to a faster rate of clinical deterioration [4].

Thus, the aim of the present study was to evaluate the method by which multi-symptom and HRQoL PROs have been validated in oncological PC settings regarding test-retest reliability.

## Methods

### Design

A systematic literature review was used.

### Eligibility criteria

The studies included in this systematic review met all of the following criteria: (1) validation study of a multidimensional quality of life instrument or a multidimensional symptom assessment instrument; (2) publication in a peer-reviewed journal; and (3) analysis of a population composed mainly of advanced cancer patients undergoing PC (or hospice care, end-of-life care, or some similar type of care).

Studies were excluded for any of the following reasons: (1) the study was not published as a full article (i.e., conference proceedings were excluded); (2) the study contained pediatric data; or (3) the publication was a duplicate publication.

### Data sources

Validation studies of PROs were retrieved from the following online databases: PubMed (1966 to June 2013), EMBASE (1980 to June 2013), PsychInfo (1806 to June 2013), CINAHL (1980 to June 2013), and SCIELO (1998 to June 2013). The Patient-Reported Outcome and Quality of Life Instrument Database (PROQOLID) [5] (http://www.proqolid.org/) and the Australian Centre on Quality of Life (ACQOL) [6] (http://www.deakin.edu.au/research/acqol/index.php) were also used to search for validation studies.

### Search strategy

Our search strategies for PubMed included the following: (1) quality of life instruments: (instrument OR questionnaire OR scale OR inventory OR checklist) AND (reliability OR test-retest OR validation OR psychometric* OR retest OR repeatability) AND (cancer OR tumor OR tumour OR carcinoma OR malignancy OR “neoplasms” [MESH]) AND “quality of life” AND (palliative care OR end-of-life OR “end of life” OR hospice OR terminal OR advanced); (2) multiple-symptom instruments: (instrument OR questionnaire OR scale OR inventory OR checklist) AND (reliability OR test-retest OR validation OR psychometric* OR retest OR repeatability) AND (cancer OR tumor OR tumour OR carcinoma OR malignancy OR “neoplasms” [MESH]) AND (symptom OR symptoms) AND (palliative care OR end-of-life OR “end of life” OR hospice OR terminal OR advanced). Searches using EMBASE, PsychInfo, CINAHL, and SCIELO were conducted by combining each of the terms used in the PubMed search strategy. With regards to the PROQOLID and ACQOL databases, references were individually screened. To identify additional papers, the reference lists of relevant articles were reviewed by one of the authors (CEP).

### Data extraction

Initial searches (titles and abstracts) were conducted independently by CPS and FT. The studies with full text available were further reviewed, the data were independently extracted by two other reviewers (CEP, CPS), and the data were verified by a third reviewer (BSRP).

A standardized data collection form was used. The data collected included study demographics (year of publication, country in which the study was conducted, language in which the instrument was administered), the name of the instrument, and information about the characteristics of the patients enrolled (age, performance status). We also collected data regarding the statistical methods employed to perform the test-retest analysis, the time frame from test to retest, the total number of patients included in the study, and the number of patients included in the reliability test-retest analysis. If the sample size for the test-retest reliability was planned a priori, the study was included only if the article stated that the patient was clinically stable. The following QoL domains were systematically extracted from each article: global, physical, psychological, social, existential/spiritual, and functional. With regard to the symptoms, we specifically analyzed the pain, fatigue, nausea, anxiety, and depression domains.

### Analytic approach

The COSMIN (COnsensus-based Standards for selection of health Measurement INstruments) checklist [7] was used to rate the methodological quality of the study designs. Because our focus was test-retest reliability, only the COSMIN Box B (reliability) was used. The “worst score counts” algorithm was used for the analysis [8]. Briefly, each item from COSMIN Box B was rated individually as “excellent”, “good”, “fair”, or “poor”, and an overall score was given by taking the lowest score of any of the items.

Because different statistical methods were used in many of the studies, a robust meta-analysis of the data was not possible. Therefore, to perform a pooled analysis, we followed the method of Terwee et al. [9] and accepted a minimum reliability threshold of 0.70 as a measure of “adequate test-retest results”. Each extracted domain was classified as the number of articles with test-retest values ≥ 0.70. The number of adequate test-retest results was associated with the evaluated outcome (HRQoL *versus* symptoms) and with the evidence provided for clinical stability, as measured by item 7 of COSMIN Box B. For this analysis, a chi-square test for linear trend was used. In addition, the time (in hours) to retest was compared between groups with adequate and non-adequate test-retest results using the Mann–Whitney *U* Test. Data are presented as median values and percentiles of 25 (P25) and 75 (P75).

The PRISMA (Preferred Reporting Items for Systematic Reviews and Meta-Analyses) guidelines for building reviews were followed during the preparation of this review (see the PRISMA checklist in the Supplementary file).

## Results

**Figure 1:**
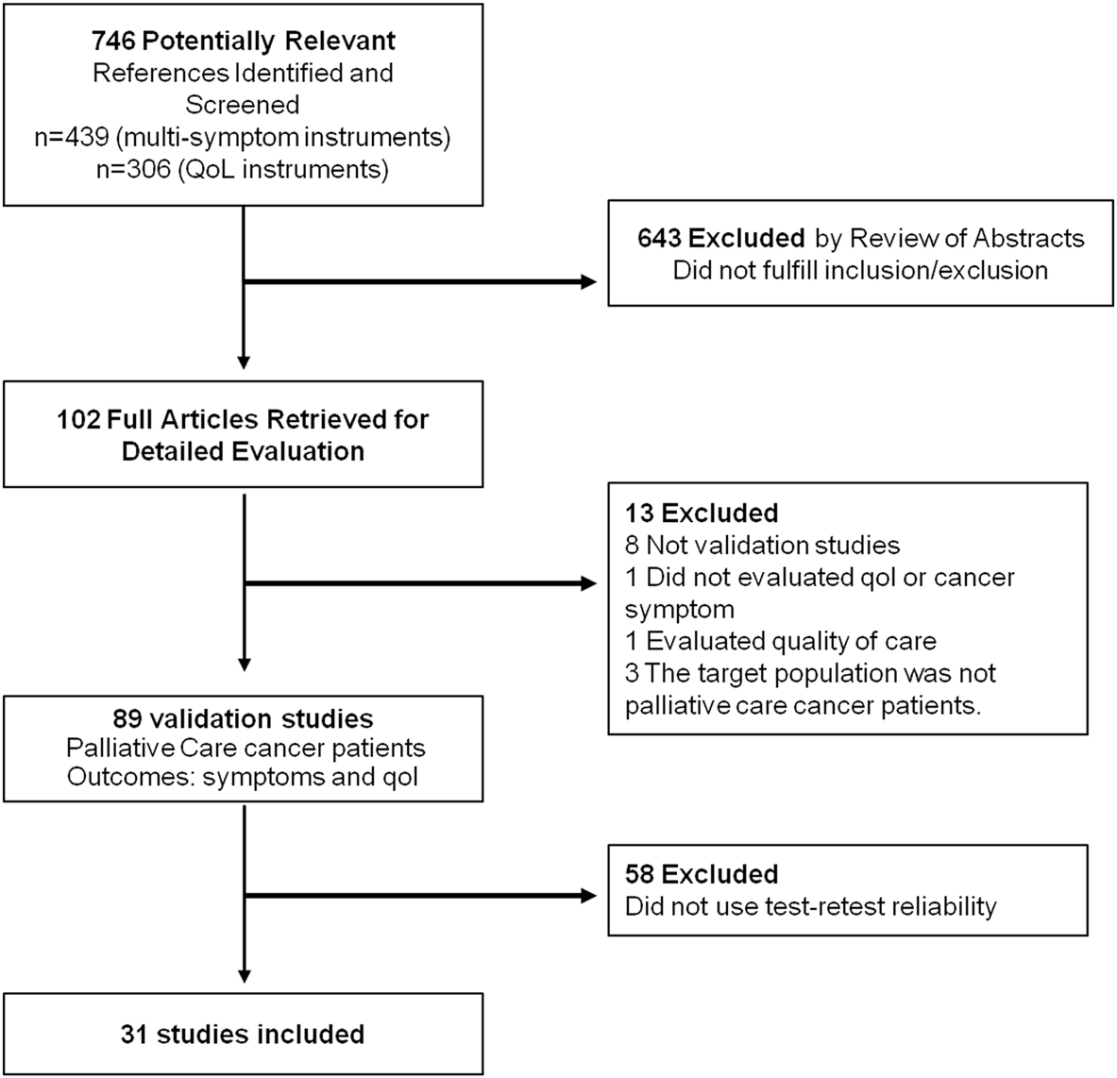
PRISMA flow diagram for search strategy.

Figure 1 summarizes the identification and selection of studies. We identified 89 articles describing validation studies of PRO that evaluated advanced cancer patients under PC. Of those, 31 (34.8%) measured test-retest reliability. Information from the included studies is detailed in Table 1.

**Table 1:** Instruments developed to assess symptoms or quality of life of cancer patients under palliative care

**Author**	**Country (language)**	**Instrument**	**No. of items**	**Population profile**	**Sample**		**Time to retest**	**Objective confirmation of clinical stability**	**Type of statistics used**
					**n (total)**	**n (retest)**			
Axelsson et al. [10]	Sweden (Swedish)	AQEL	22	ACP. Median age = 67.5 years, mean and median KPS = 62.3 and 70, respectively.	71	30	3 days	Not described	Spearman Rank correlation
Cohen et al. [11]	Canada (English)	MQOL	16	ACP. Could be receiving chemotherapy. Mean age: 59 years). Life expectancy greater than 2 weeks.	100	49-59	2 days	Yes	ICC
Llobera et al. [12]	Spain (Spanish)	HRCA-QL index	5	APC (end-of-life). Median survival = 59 days.	200	39	1 month (mean)	Yes	ICC
Mystakidou et al. [13]	Greece (Hellenic)	PLQI	28	Patients with symptomatic incurable cancer. Off anticancer treatment for ≥ 3 months. All patients classified as ECOG-PS ≥ 2 (ECOG-PS3 = 68%).	120	120	7 days	No	Spearman Rank correlation
Steinhauser et al. [14]	USA (English)	QUAL-E	25	Terminal CHF, ESRD, COPD, and stage IV cancer (56%).	248	135-170	7 days	Yes	ICC
Lopes Ferreira et al. [15]	Portugal (Portuguese)	POS	10	ACP (life expectancy < 1 year), non-terminal, many of them undergoing chemotherapy.	109	30	7 days	No	Pearson correlation
Suárez-del-Real et al. [16]	Mexico (Spanish)	QLQ-C15-PAL	15	ACP undergoing exclusive palliative care. KPS < =70% = 78%.	83	30	15.7 days (mean)	No	Repeated measures ANOVA
Leppert et al. [17]	Poland (Polish)	QLQ-C15-PAL	15	ACP undergoing palliative care.	129	129	7 days	No	Spearman Rank correlation
Kim et al. [18]	Korea (Korean)	MQLS	32	Mortality rate during study = 80%, ECOG-PS > = 3 = 71.2%.	70	54-64	3 hours and 7 days	No	ICC
Kim et al. [19]	Korea (Korean)	HQLS	43	Advanced cancer patients under exclusive palliative care. ECOG-PS3/4 = 64.2%.	180	88	1-2 weeks	No	Not described
Serra-Prat et al. [20]	Spain (Spanish)	POS	10	ACP under palliative care. Mean KPS = 50.93%.	200	Not described	1 week	Yes	ICC
Eisenchlas et al. [21]	Argentina (Spanish)	POS	10	ACP under palliative care. ECOG-PS 3/4 = 58%.	65	24	2 days	No	Weighted kappa agreement
Guo et al. [22]	USA (English)	Brief Hospice Inventory	17	Geriatric population (mean age = 78 years, 83% ≥ 70 years) 47% Advanced cancer under hospice care in 47%.	145	63	1 week	No	Not described
Lo et al. [23]	China (Chinese)	MQOL	16	Incurable ACP.	462	20	2 days	No	ICC
Shahidi et al. [24]	Iran (Persian)	MQOL	16	Mean KPS = 74.8%. Any type of incurable cancer with an estimated life expectancy of < 12 months was considered for the study.	61	Not clearly stated	24-72 hours	No	Pearson correlation
Harding et al. [25]	South Africa and Uganda (most spoke IsiZulu^1^)	APCA African Palliative Outcome Scale	10	Hospice patients, including ACP. Limited self-care and completely disabled = 41.7%.	682	307	21.2 hours (mean)	No	ICC
Hearn et al. [26]	England and Scotland (English)	POS	10	ACP (98%) under palliative care. Limited activity or disabled = 47.1%.	168	34	3-7 days	Yes	Weighted kappa agreement
Wilson et al. [27]	Canada (English)	SISC	13	ACP under palliative care. Median survival = 46 days.	68	46	1-3 days	No	Pearson correlation
Agra et al. [28]	Spain (Spanish)	RSCL	39	ACP under palliative care. Terminal illness (life expectancy < 6 months). KPS < 70% in 65.2%.	118	116	24 hours	No	ICC
Chang et al. [29]	USA (English)	ESAS	10	ACP under palliative care. Mostly elderly male patients.	233	19-23	1 day and 1 week	No	Spearman Rank correlation
Stiel et al. [30]	Germany (German)	MIDOS	12	ACP under palliative care. ECOG-PS 3/4 = 56.6%.	60	60	1 day	No	Pearson correlation
Moro et al. [31]	Italy (Italian)	ESAS	10	ACP under exclusive palliative care. In-patients, KPS ≤ 40 = 75%. Median survival = 35 days.	241	60	24-48 hours	No	ICC
Mystakidou et al. [32]	Greece (Greek)	MDASI	19	ACP under palliative care. ECOG-PS 3/4 = 63%.	150	100	3 days	No	Pearson correlation
Pautex et al. [33]	Swiss (French)	ESAS	10	ACP under palliative care. Mean age = 72 years.	42	42	1 day	No	Paired *t* test
Stiel et al. [34]	Germany (German)	HOPE-SP-CL	16	ACP under palliative care; inpatients.	31,055	332-472	7 days	No	Spearman Rank correlation
Carvajal et al. [35]	Spain (Spanish)	ESAS	10	ACP under palliative care. KPS ≤ 70% = 45.5%.	171	146	0-6 hours	No	Spearman Rank correlation and Lin’s Concordance test
Radbruch et al. [36]	Germany (German)	MIDOS	12	ACP under palliative care. ECOG-PS 3/4 = 81.2%.	128	76	1-6 days	Yes	Pearson correlation
Kwon et al. [37]	Korea (Korean)	ESAS	10	Mostly symptomatic ACP undergoing active antineoplastic treatment.	162	162	2-4 hours	No	Pearson correlation
Aoun et al. [38]	Australia (English)	SAS	7	ACP under palliative care.	572	60	2 hours	Yes	ICC
Pereira et al. [39]	Portugal (Portuguese)	FACT-G	28	ACP under PC (end-of-life). Inpatients and outpatients. Performance status not described.	346	27	8-9 days	No	Pearson correlation
Sterkenburg et al. [40]	Canada (English)	MQLS	32	Most were ACP under palliative care. Mean survival = 49.56 days.	84	73	3 hours and 7 days	No	ICC

*USA* United States of America, *ICC* intraclass correlation coefficient, *CHF* chronic heart failure, *ANOVA* Analysis of variance, *ESRD* end stage renal disease, *COPD* chronic obstructive pulmonary disease, *AQEL* Assessment of Quality of Life at the End of Life, *MQOL* McGill Quality of Life Questionnaire, *HRCA-QL index* Hebrew Rehabilitation Centre for Aged Quality of Life Index, *PLQI* The Palliative Care Quality of Life Instrument, *QUAL-E* Quality of Life at the End of Life, *POS* Palliative Care Outcome Scale, *QLQ-C15-PAL* The European Organization for Research and Treatment of Cancer (EORTC) QLQ-C15-PAL, *MQLS* McMaster Quality of Life Scale, *HQLS* Hospice Quality of Life Scale, *SISC* Structured Interview for Symptoms and Concerns, *RSCL* Rotterdam Symptom Check List, *ESAS* Edmonton Symptom Assessment System, *MIDOS* Minimal Documentation System, *MDASI* MD Anderson Symptom Inventory, *HOPE-SP-CL* Multidimensional symptom and problem checklist, *SAS* Symptom Assessment Scale, *FACT-G* Functional Assessment of Cancer Therapy- General. ^1^ IsiZulu (49.6%), English (15.3%), isiXhosa (12.8%), Luganda (6.4%), SeSotho (6.3%), Runyoro (5.4%), Runyankole (3.9%), and SeTswana (0.4%).^2^ 7 for the patients and 3 for the caregivers.

### Methodological quality of the studies

**Figure 2:**
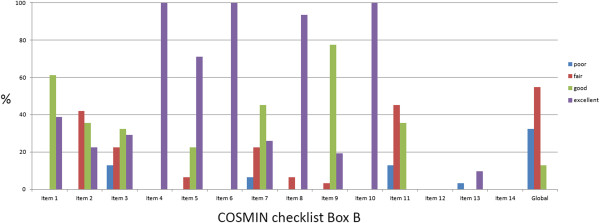
Quality criteria of the included studies according to the COSMIN checklist.

Two authors (CEP, EMB) classified the articles according to the COSMIN guidelines; the percentage of agreement between coders was 85.3% (Cohen’s Kappa coefficient = 0.764). Any disagreements in interpretation were resolved by a discussion with a third author (BSRP). There were 4 (12.9%) [11,25,28,40], 17 (54.8%) [10,12-15,18,20,24,26,27,30-32],[34,36-38], and 10 (32.2%) [16,17,19,21-23,29,33,35,39] articles classified as good, fair, and poor, respectively, with regards to the overall quality criteria. No article was classified as excellent according to the aforementioned criteria. The global quality classification per item is described in Figure 2.

### Sample sizes

A total of 29 studies (29 of 31; 93.5%) [10-19,21-23,25-40] described the number of patients submitted to the test-retest analysis. Of those, the median (P25-P75) number of patients included was 60 (32–119). The majority of the studies (24 of 29; 82.8%) [10-12,14-16,18,19,21-23,25-29,31,32],[34-36,38-40] included fewer than the total number of patients for the reliability analysis. Overall, 53.8% (95% CI 19.6%-87.9%) of the total number of analyzed patients were used for the test-retest reliability analysis. Only 2 articles [28,37] described the sample size calculation for the test-retest analysis. One study [35] used a reference from others recommending that at least 50 patients should be used for this type of statistical analysis.

### Time to retest

The time interval to retest was clearly stated in all of the included studies [10-40]. The median (P25-P75) time was 72 (27–168) hours. The median (P25-P75) time intervals for the retest were 24 (3.25-60) hours and 168 (48–204) hours for the symptom and HRQoL validation studies, respectively (p = 0.001).

### Confirmation of clinical stability

Of the 31 analyzed articles, 10 (10 of 31, 32.3%) [11-15,20,21,26,36,38] clearly stated that only clinically stable patients were submitted to the retest. The confirmation of clinical stability in accordance with the COSMIN checklist was associated with adequate results for the test-retest analysis regarding both pain and global HRQoL scores (p < 0.05, Table 2). Of those studies, 6 used 1 or more of the following objective criteria to define a stable condition: patient perception of change (n = 2) [11,20]; stable doses of opiates (n = 1) [36] or lack of a new medication for symptom treatment (n = 2) [14,38]; emergency department visit and/or hospitalization (n = 1) [14]; and change in Performance Status or in daily living activities (n = 1) [12].

**Table 2:** Association between the test-retest reliability values and the evidence of clinical stability

**Test-retest values**	**Were patients stable in the interim period on the construct to be measured?**				***P*** *
	**Poor**	**Fair**	**Good**	**Excellent**	
	**N (%)**	**N (%)**	**N (%)**	**N (%)**	
Global HRQoL scores					0.015
< 0.70	2 (18.2)	5 (45.5)	4 (36.4)	0 (0)	
≥ 0.70	0 (0)	1 (12.5)	5 (62.5)	2 (25.0)	
Pain scores					0.031
< 0.70	2 (18.2)	3 (27.3)	3 (27.3)	1 (9.1)	
≥ 0.70	0 (0)	1 (11.1)	4 (44.4)	4 (44.4)	

*HRQoL* health-related quality of life.

*p-value for linear trend.

### Scores of retest

In the present review, we chose to perform statistical comparisons only for the pain and global HRQoL scores because they were the most commonly described domains in the selected studies (Table 3). Taking into consideration a set value of ≥ 0.70 as an adequate result in the test-retest analysis, 50% (9 of 18, 50%) [13,20,25,29,32,35-38] and 45% (9 of 20, 45%) [10-12,18,23-25,32,40] of the studies with pain and global HRQoL values, respectively, were considered adequate.

**Table 3:** Number of studies with adequate and non-adequate test-retest values

**Evaluated domains**	**Number of studies**	**Test-retest values**	
		**<0.70**	**≥0.70**
		**n (%)**	**n (%)**
Global HRQoL	19	11 (57.9)	8 (42.1)
Emotional	8	1 (12.5)	7 (87.5)
Physical	5	1 (20)	4 (80)
Social	6	2 (33.3)	4 (66.7)
Functioning	3	0 (0)	3 (100)
Existential	6	1 (16.7)	5 (83.3)
Global symptoms	5	2 (40)	3 (60)
Pain	18	9 (50)	9 (50)
Nausea	12	5 (41.6)	7 (58.3)
Fatigue	11	6 (54.5)	5 (45.5)
Anxiety	10	6 (60)	4 (40)
Depression	9	4 (44.4)	5 (55.6)

*HRQoL* health-related quality of life.

There was a non-significant trend favoring shorter time intervals for the retest in the studies with adequate results for the retest statistical analysis (value ≥ 0.70) in comparison with those with non-satisfactory results (value <0.70) (Table 4).

**Table 4:** Median values of time intervals of studies with non-adequate (<0.70) and adequate (≥0.70) test-retest values

**Evaluated domains**	**Number of studies**	**Time interval (hours) Median (Q1-Q3)**		p-value*
		**Test-retest < 0.70**	**Test-retest ≥ 0.70**	
Global HRQoL	19	168 (36–168)	60 (36.75-206)	0.565
Emotional	8	168^1^	168 (48–204)	-
Physical	5	168^1^	108 (30–195)	-
Social	6	186^2^	108 (48–195)	-
Functioning	3	-	168^3^	-
Existential	6	120^1^	168 (48–232)	-
Global symptoms	5	25.75^2^	48^3^	-
Pain	18	120 (42–168)	48 (3–168)	0.262
Nausea	12	108 (33–168)	24 (3–72)	0.146
Fatigue	11	48 (24–168)	3 (2.5-120)	0.184
Anxiety	10	72 (36–168)	48 (8.25-144)	0.443
Depression	9	102 (27–168)	60 (18.75-96)	0.587

*HRQoL* health-related quality of life.

*Mann–Whitney test.

^1^Only one study. ^2^Only two studies. ^3^Only three studies.

Three studies [18,29,40] compared 2 different time frame intervals for the retest. Two of them [18,40] measured global HRQoL 3 hours and 7 days after the first evaluation; the test-retest results were 0.84-0.93 and 0.63 at 3 hours and 7 days, respectively. The other validation study [29] evaluated cancer symptoms using the Edmonton Symptom Assessment System (ESAS) scale. That study found higher test-retest values for shorter time intervals, with the exception of the symptom of fatigue (Table 5).

**Table 5:** Test-retest reliability scores measured at two different time intervals from the first evaluation

**Author (year)**	**Time to retest**	**Scores**					
		**Global HRQoL**	**Pain**	**Nausea**	**Fatigue**	**Anxiety**	**Depression**
Kim et al. [18]	3 hours	0.93	NA	NA	NA	NA	NA
	7 days	0.63	NA	NA	NA	NA	NA
Chang et al. [29]	24 hours	NA	0.79	0.58	0.39	0.62	0.81
	7 days	NA	0.75	0.31	0.65	0.35	0.54
Sterkenburg et al. [40]	3 hours	0.84	NA	NA	NA	NA	NA
	7 days	0.63	NA	NA	NA	NA	NA

*NA* not evaluated.

### Statistical methods used

Of those instruments with continuous scores (n = 29), 11 (11 of 29, 37.9%) [11,12,14,18,20,23,25,28],[31,38,40] evaluated the test-retest reliability using the intraclass correlation coefficient, and 14 (14 of 29, 48.3%) [10,13,15,17,24,27,29,30],[32,34-37,39] performed some type of correlation analysis such as Spearman’s (n = 6) or Pearson’s (n = 8) test. Interestingly, for those studies in which the intraclass correlation coefficient (ICC) method was used, none described the statistical model or formula used; therefore, no study could be classified as “excellent” according to item 11 in Box B of the COSMIN guidelines. Two studies used paired analysis (repeated measures of analysis of variance [ANOVA], n = 1; paired *t* test, n = 1) to evaluate test-retest reliability [16,33]. Three studies with ordinal score instruments calculated the weighted kappa statistic [20,21,26]. Two studies did not describe the type of statistics used [19,22].

## Discussion

In this study, we investigated the methods by which validation studies of PRO have been performed in the PC setting, particularly with regards to test-retest reliability. In general, the methodological quality of this psychometric property was investigated poorly to fairly; according to the COSMIN checklist, only 12.3% of the studies were considered of good quality, and none were considered of excellent quality. In addition, we highlighted the importance of verifying the clinical stability of advanced cancer patients before performing the retest. Based on our results, clinical stability is even more important for test-retest reliability than the accurate definition of the time interval at which the retest is performed.

In our review, we identified 89 validation studies that included cancer symptoms and/or HRQoL as outcome variables. Of those, only 31 (34.8%) evaluated the test-retest reliability. As the test-retest reliability is an essential psychometric property to be measured in validation studies, we hypothesize that researchers are not systematically measuring it because of the instability of advanced cancer patients. Overall, half of the evaluated test-retest reliability scores were classified as inadequate when 0.70 was used as the threshold value [9]. The pressure experienced by scientific researchers to publish positive results [41] may also explain why only 34.8% of validation studies measured the test-retest reliability. Furthermore, it is possible that inappropriate test-retest values were omitted from some publications.

It is essential to accurately estimate the sample size prior to beginning a study. An insufficient sample size might not detect true differences, which might lead to unreliable results. Conversely, an excessive sample size may produce unnecessary financial losses and ethical concerns regarding futile exposure of study participants [42]. With regards to test-retest reliability analysis, we observed that determining an adequate sample size is not a common practice because only 2 studies [28,37] described performing a sample size calculation prior to the study. Overall, the median number of included patients for test-retest analysis was 60, which represents 53.8% of the total number of included patients. One study [35] justified the sample size by citing a rule of thumb suggesting that 50 patients would be sufficient for the analysis [43,44].

A basic concept regarding test-retest reliability is the need to retest clinically stable patients [45]. The retest of advanced cancer patients is challenging because they are in a dynamic phase of their disease in which symptoms and functionality are prone to decline quickly. The retest of a clinically unstable patient may incorrectly define a PRO as a non-reliable tool. Our results confirm the importance of verifying the clinical stability of the patients before retesting. In addition, our review described the objective criteria used by some studies to define a stable condition.

The definition of an adequate between-assessment time gap for the retest is of the utmost importance. An insufficient time period might allow respondents to recall their first answers, and a longer interval might allow for a true change of the construct to occur [2,45]. The appropriate time interval depends on the construct to be measured and the target population [46]; however, approximately 2 weeks is often considered generally appropriate [47]. Nevertheless, the time interval over which to retest advanced cancer patients under PC is still a matter of debate. Some authors have considered retesting advanced cancer patients at least 3 days apart as a measure of responsiveness but not as a measure of test-retest reliability [4].

In fact, because of concerns about reassessing an unstable patient, some authors (n = 7) reapplied the questionnaires at very short intervals (i.e., less than 24 hours). Jim et al. [48] investigated daily and intraday changes in the fatigue, depression, sleep, and activity scores in a cohort of cancer patients undergoing chemotherapy. Significant changes were observed over time. Additionally, Dimsdale et al. [49] investigated cancer-related fatigue every hour for 72 consecutive hours and observed a diurnal variation in fatigue. HRQoL, on the other hand, is a multidimensional construct that encompass physical, psychological, social, and spiritual domains. In general, instruments that measure HRQoL use recall periods of 7 days. Although HRQoL is not commonly assessed on a daily basis, it is expected to behave stably over a few days, especially the social, existential, and global domains. Consequently, we observed that multi-symptom instruments are generally retested within a shorter time frame than HRQoL instruments.

There was a trend of shorter time periods in the adequate test-retest reliability results when compared with the scores with inadequate results (less than 0.7). One reason contributing to the non-significant results might be the large interquartile range for some of the domains; since few studies were analyzed, there was insufficient statistical power for further conclusions. Three studies [18,29,40] evaluated the retest reliability at 2 different time points (< 24 hours and 1 week after the first evaluation); in general, a lower time interval was associated with a better retest analysis result. Considering the median time interval used in the studies with adequate test-retest results, in addition to the findings from studies that used two different time intervals for the retest, we can recommend that patients under palliative care for advanced cancer should be retested somewhere around 24 to 48 hours later when evaluating cancer symptoms and 2 to 7 days later when assessing HRQoL. However, we believe that the most important factor is not the time itself but rather confirmation of clinical stability before retesting patients.

As mentioned previously, we concluded that the test-retest reliability analysis was of low quality according to the COSMIN checklist. Other studies using the same guidelines but not the same population have yielded similar results [50-52]. The most troublesome question in our review was item 11 (“for continuous scores: was an intraclass correlation coefficient calculated?”), with 62% of studies classified as poor to fair quality. The preferred test-retest reliability statistic depends on the type of response options. In our review, the majority of the studies evaluated continuous scores. In these cases, the ICC [31] is the preferred statistic [46,47,53]. Moreover, the use of correlation coefficients (Pearson’s and Spearman’s tests) is not adequate because they do not include a consideration of systematic error [46]. In the present study, 18 of 29 studies evaluating continuous scores used a correlation analysis, but they did not evaluate the agreement by using the ICC. Six different versions of ICC can be used depending on various assumptions, and 4 of those are subdivided into consistency or absolute agreement, yielding a total of 10 different ICC calculations [54]. The choice of the correct index has a highly significant impact on the numerical value of the ICC [53]. Even in those studies that correctly used the ICC, none stated the version of the ICC used.

This study has some limitations. Because the studies evaluated test-retest reliability using different statistics, we could not perform a robust meta-analysis. Therefore, we decided to use 0.7 as the threshold for adequate results on test-retest reliability to perform a pooled data analysis. However, the categorization of the test-retest results as a function of a predefined cut-off point may be considered an inadequate simplification. Another limitation is that we did not include in the systematic review other instruments developed to assess only one symptom (fatigue or pain scales, for example). In addition, we did not include abstracts from meetings because it would be difficult to extract the necessary data.

## Conclusions

In conclusion, we determined that test-retest reliability has been infrequently and poorly evaluated in validation studies of PRO assessing advanced cancer patients under PC. Multi-symptom instruments were retested over a shorter time interval when compared to HRQoL. The confirmation of clinical stability was an important factor in our analysis, and we suggest that special attention to this parameter is required when designing a PRO validation study that includes advanced cancer patients under PC.

## Competing interests

The authors declare that they have no competing interests.

## Authors’ contributions

CEP and BSRP conceived the study. CPS and FT identified the studies. CEP, CPS, and BSRP extracted the data. CEP and EMB evaluated the quality of the studies according to the COSMIN checklist. CEP and ECC performed the analyses. CEP, ECC, RVML, EMB, and BSRP drafted the manuscript. All authors read and approved the final manuscript.

## Pre-publication history

The pre-publication history for this paper can be accessed here:

http://www.biomedcentral.com/1471-2288/14/8/prepub
